# Transcranial Temporal Interference Stimulation: A Brief Review of Architectures, Circuits, and Application Challenges

**DOI:** 10.1109/OJEMB.2026.3689259

**Published:** 2026-04-30

**Authors:** Arnau Diez-Clos, Simona Losacco, Alejandro D. Fernndez Schrunder, Stphane Emery, Friedhelm C. Hummel, Mahsa Shoaran, Komail Badami

**Affiliations:** CSEM 548509 8005 Zurich Switzerland; Institute of Electrical and Micro Engineering, cole Polytechnique Fdrale de Lausanne 1015 Lausanne Switzerland; Neuro-X Institutecole Polytechnique Fdrale de Lausanne 1202 Geneva Switzerland; CSEM 548509 8005 Zurich Switzerland; Neuro-X Institutecole Polytechnique Fdrale de Lausanne 1015 Geneva Switzerland; Clinique Romande de RadaptationNeuro-X Institute, cole Polytechnique Fdrale de Lausanne Valais 1951 Sion Switzerland; University of Geneva 27212 1204 Geneva Switzerland; Institute of Electrical and Micro Engineeringcole Polytechnique Fdrale de Lausanne 1015 Lausanne Switzerland; Neuro-X Institutecole Polytechnique Fdrale de Lausanne 1202 Geneva Switzerland

**Keywords:** Deep brain neuromodulation, integrated circuits, neuromodulation systems, noninvasive stimulation, temporal interference stimulation

## Abstract

Noninvasive neuromodulation methods are promising for treating neurological diseases, but generally have limited ability to selectively target deep brain structures such as the basal ganglia or hippocampus. Transcranial temporal Interference Stimulation (tTIS) overcomes this limitation by superimposing high-frequency electric fields to create low-frequency amplitude-modulated envelopes that can preferentially target deep brain structures. This review explores the operation principles and recent advances of tTIS aimed at improving its spatial accuracy and versatility. Additionally, we analyze the key aspects of tTIS system design, including waveform generation, high-voltage-compliant output stage, and real-time charge balancing, which collectively enable safe and efficient stimulation delivery. We further discuss key challenges such as safety, standardization, and long-term efficacy, and outline future directions toward personalized tTIS.

## I. Introduction

**Fig. 1.**
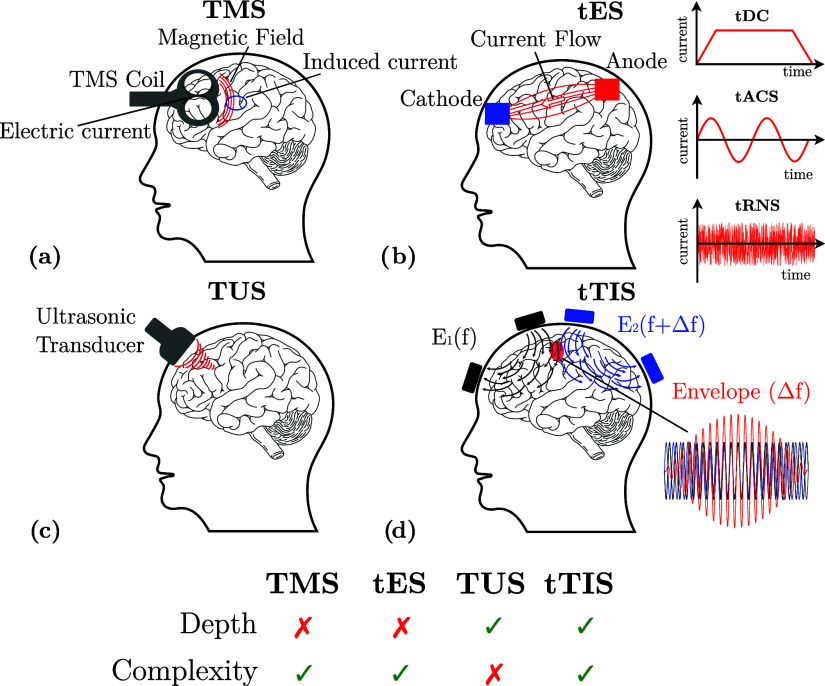
Comparison of noninvasive neuromodulation techniques: (a) TMS, (b) tES, (c) TUS, (d) tTIS.

The modulation of neural activity in deep brain regions such as the basal ganglia, thalamus, or hippocampus has traditionally required invasive methods. Attempts to target those regions non-invasively are hindered by a steep depthfocality tradeoff, which leads to unwanted stimulation of overlying structures [1], [2]. As a result, conventional noninvasive techniques such as transcranial magnetic stimulation (TMS) (Fig. 1(a)) and transcranial electrical stimulation (tES) (Fig. 1(b)) can reach deeper regions only with limited selectivity and therefore predominantly modulate cortical areas. Transcranial focused ultrasound (TUS) (Fig. 1(c)) offers a noninvasive method for targeting deep brain regions with high spatial precision. However, its widespread adoption is limited by system complexity, as accurate targeting generally relies on individualized anatomical imaging and skull modeling, often combined with dedicated planning and navigation tools [3]. In addition, heating of the targeted tissue remains a potential side effect, and the underlying mechanisms of the technique are not yet fully understood [4].

Temporal Interference Stimulation (TIS) is a promising neuromodulation technique that, when applied transcranially (tTIS), can improve the depthfocality trade-off in brain stimulation by generating deep amplitude-modulated electric fields [5]. By delivering two high-frequency electric fields oscillating in the kHz range with a small frequency offset ($\Delta f$) (Fig. 1(d)), it is possible to create a low-frequency amplitude-modulated (AM) envelope that can be focused deep within the brain.

The carrier frequencies used in tTIS are generally above the range that efficiently evokes direct neuronal firing. Nevertheless, the low-frequency amplitude modulation arising from the interference of the two fields can interact with neuronal and network dynamics, even when an unmodulated kHz carrier alone produces little central effect. The mechanisms underlying this interaction remain under investigation. Proposed explanations include subthreshold modulation of membrane polarization and spike timing [5], [6], as well as nonlinear membrane processes such as ion-channel-mediated rectification [7]. Similar to other tES techniques, tTIS is therefore generally thought to influence neuronal membrane polarization and spike timing rather than directly evoke firing. tTIS has shown promising results in targeting deep brain structures in humans, including the striatum [8], [9], hippocampus [10], [11], [12] and globus pallidus [13], while maintaining favorable tolerability and safety profiles [14], [15]. These findings highlight the potential of tTIS for treating neurological and psychiatric conditions such as epilepsy, stroke, Parkinsons disease, Alzheimers disease and depression by targeting deep brain regions involved in their pathophysiology.

This paper provides a concise overview of the operating principles of tTIS in Section II. Section III addresses system-level design innovations, while Section IV details the circuit-level implementation of tTIS stimulators. Key challenges related to safety, calibration, and long-term efficacy are discussed in Section V. Future directions for tTIS hardware and clinical translation are outlined in Section VI. Finally, the conclusions are presented in Section VII.

## II. tTIS Operating Principles

tTIS achieves deep brain neuromodulation by simultaneously injecting two sinusoidal currents with frequencies $f_{1}$ and $f_{2}$ and amplitudes $A_{1}$ and $A_{2}$ [5]:

\begin{align*}
 I_{1}(t) = A_{1} \sin (2\pi f_{1} t), \quad I_{2}(t) = A_{2} \sin (2\pi f_{2} t). \tag{1} 
\end{align*}

Within neural tissue, these currents induce the corresponding electric fields $\vec{E}_{1}(\vec{r})$ and $\vec{E}_{2}(\vec{r})$ at the location $\vec{r}=(x,y,z)$, with an angle $\alpha$ between them. The superposition of these fields results in an amplitude-modulated electric field (Fig. 1(d)) given by:

\begin{align*}
 \vec{E}(\vec{r}, t) = \vec{E}_{1}(\vec{r})\,\sin (2\pi f_{1} t) +\vec{E}_{2}(\vec{r})\,\sin (2\pi f_{2} t). \tag{2} 
\end{align*}

The field oscillates at the carrier frequency $f_{c} = \frac{f_{1} + f_{2}}{2}$ with an envelope modulation frequency $\Delta f = |f_{1} - f_{2}|$. The maximal envelope modulation amplitude $\vec{E}^{\max }_{\mathrm{AM}}(\vec{r})$ depends on the magnitudes of $E_{1}(\vec{r})$, $E_{2}(\vec{r})$, and the angle $\alpha$. For $|E_{1}(\vec{r})| < |E_{2}(\vec{r})|$ and $\alpha < 90^\circ$, it is computed as [5], [20]:

\begin{align*}
 &\bigl |\vec{E}^{\max }_{\mathrm{AM}}(\vec{r})\bigr | =\\
 & {\begin{cases}2\,|E_{2}(\vec{r})|, & \text{if } |E_{2}(\vec{r})| \!\!<\! |{E}_{1}(\vec{r})|\cos (\!\alpha), \\[4pt]
 \frac{2\,\bigl \vert E_{2}(\vec{r})\times \bigl (E_{1}(\vec{r})-E_{2}(\vec{r})\bigr)\bigr \vert }{\bigl \vert E_{1}(\vec{r})-E_{2}(\vec{r})\bigr \vert }, & \mathrm{otherwise.} \end{cases}} \tag{3} 
\end{align*}

Equation (3) shows that the modulation amplitude is maximized when the two fields are aligned ($\alpha =0^\circ$) and significantly reduced when they are perpendicular ($\alpha =90^\circ$). Furthermore, the maximum envelope amplitude occurs where the magnitudes of the two fields are equal. This causes the maximum stimulation region to shift toward the electrode injecting the lower current, as the stronger field from the higher-current electrode attenuates until it balances the weaker field. By adjusting the current ratio between electrode pairs, the stimulation focus can be steered without repositioning the electrodes, highlighting a key advantage of tTIS.

## III. tTIS Architectures and Modulation Strategies

**Fig. 2.**
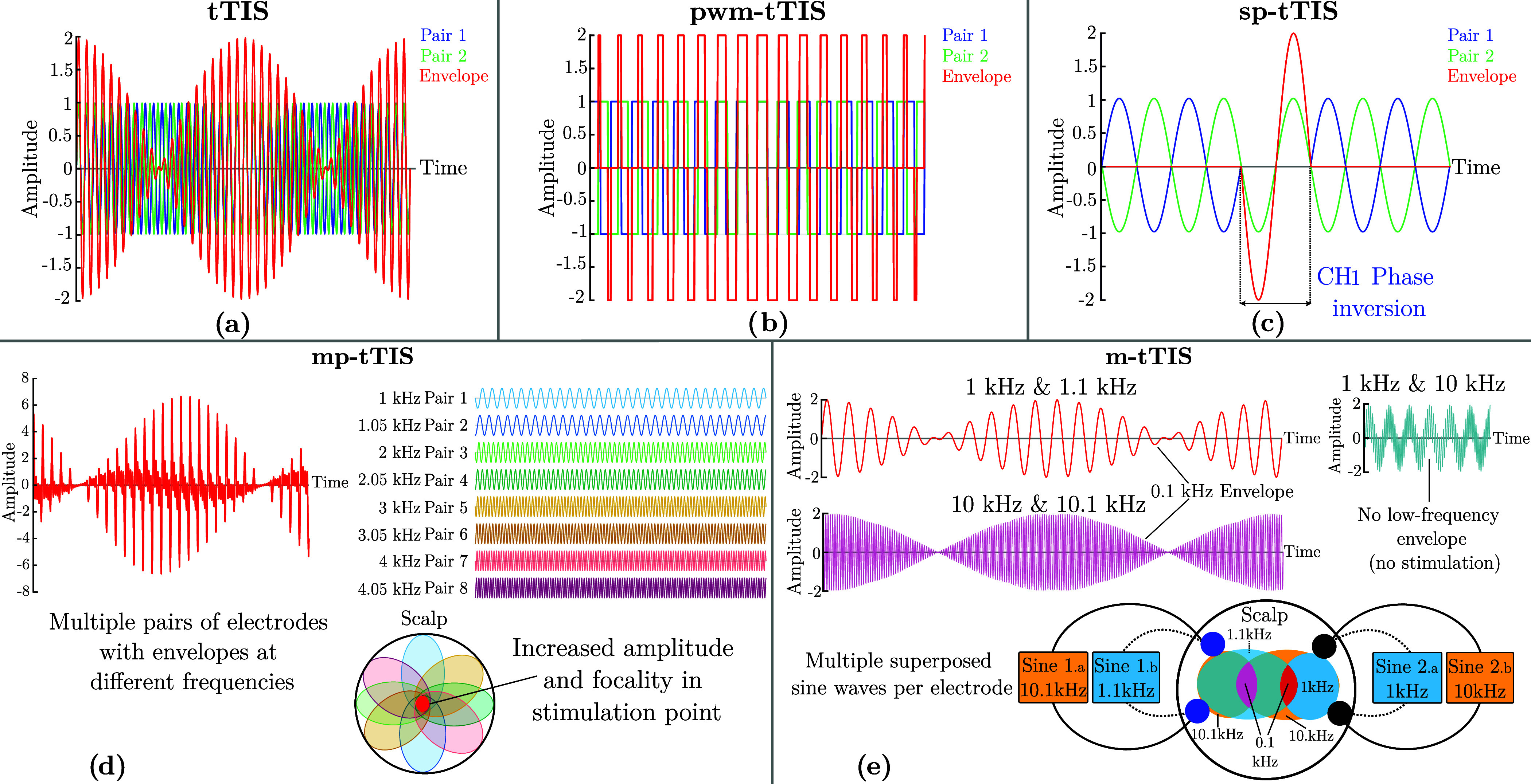
tTIS system design innovations: (a) standard tTIS, (b) pwm-tTIS, (c) sp-tTIS, (d) mp-tTIS, and (e) m-tTIS. The red (or purple in (e)) waveforms represent low-frequency envelopes resulting from the interference of high-frequency carriers (each normalized to amplitude 1). Although ideal sine waves are illustrated, actual waveforms in biological tissue are subject to distortion and attenuation. Adapted from [16], [17], [18], [19].

Researchers have developed novel tTIS architectures and modulation strategies to enhance the precision and flexibility of tTIS neuromodulation, offering potential improvements over standard tTIS (Fig. 2(a)).

### A. Pulse-Width Modulated tTIS

Pulse-width modulated tTIS (pwm-tTIS) employs two high-frequency biphasic square waveforms with constant amplitude rather than sinusoidal ones (Fig. 2(b)). Their interference generates a biphasic square waveform with frequency $f = \frac{f_{1} + f_{2}}{2}$ and a pulse width that varies periodically at the difference frequency $\Delta f = |f_{1} - f_{2}|$ (e.g., in [16] with $f_{1}$ 2000 Hz and $f_{2}$ 2010 Hz, the resulting waveform oscillates at 2005 Hz and is pulse-width modulated at 10 Hz). With the envelope having a fixed amplitude, it leverages the neural membranes low-pass filtering properties to convert the pulse-width modulated field into an amplitude-modulated membrane potential [16]. In a mouse model, pwm-tTIS has been shown to effectively modulate neural activity, achieving efficiency comparable to, and in some cases exceeding, that of conventional tTIS [16].

### B. Single-Pulse and Phase-Modulation tTIS

In tTIS, the width of the AM envelope is determined by the difference frequency $\Delta f$, which controls the duration of stimulation. Single-pulse tTIS (sp-tTIS) and phase-modulating tTIS (pm-tTIS) enhance spatial and temporal resolution by enabling direct control of the envelope width regardless of the stimulation frequency. Both methods use phase-shift keying modulation, combining carrier signals with identical frequency but opposite phase to create the desired burst patterns. sp-tTIS delivers only a single cycle of the carrier signal per burst (Fig. 2(c)), whereas pm-tTIS includes multiple periods within each burst [17], [21]. The ability to modulate phase rather than frequency enables more precise shaping of the temporal profile of the interference field [21]. Such control can benefit applications requiring high temporal resolution. However, the dynamics of these systems are highly susceptible to phase variations. Furthermore, experimental validation, particularly in human trials, remains limited and further research is needed to fully assess its clinical value.

### C. Multipolar tTIS

Multipolar tTIS (mp-tTIS) employs multiple independent electrode pairs to generate several high-frequency electric fields that constructively interfere to create a very focal envelope (Fig. 2(d)). By maintaining at least a 1 kHz difference between carrier frequencies, mp-tTIS avoids generating additional low-frequency envelopes from interactions between different carriers. Computational modeling and preclinical validation in mice and non-human primates suggest that mp-tTIS can improve focality while maintaining target intensity, with less than 1 of the brain receiving a higher envelope exposure than the target region, compared to 20 in conventional tTIS [18]. Additionally, by distributing current across multiple electrode pairs, mp-tTIS can improve focality and increase target modulation amplitude without relying on a single pair to deliver all the required current [17], [18].

### D. Multi-Point tTIS

Multi-Point tTIS (m-tTIS) enables simultaneous stimulation of multiple deep brain regions by driving each electrode with different frequency currents (e.g., 1 kHz with 1.01 kHz and 10 kHz with 10.01 kHz in Fig. 2(e)) [19]. This approach creates independent stimulation foci without requiring additional electrode pairs. The location of each focus can be independently controlled by modulating the relative current ratios, as confirmed through geometric models, MRI-based human head models, and tissue phantom experiments. This approach offers a solution for modulating multiple nodes within deep brain networks [19].

## IV. tTIS Integrated Circuit Implementation

Early tTIS implementations relied on discrete components, significantly increasing system size [22], [23], [24]. Application-Specific Integrated Circuits (ASICs) have since been developed for peripheral-nerve TIS [25], [26], [27], and more recently extended to tTIS [28]. Nevertheless, the field remains in its early stages, and further development is needed to achieve compact, power-efficient, and scalable hardware suitable for portable and wearable deep-brain stimulation.

**Fig. 3.**
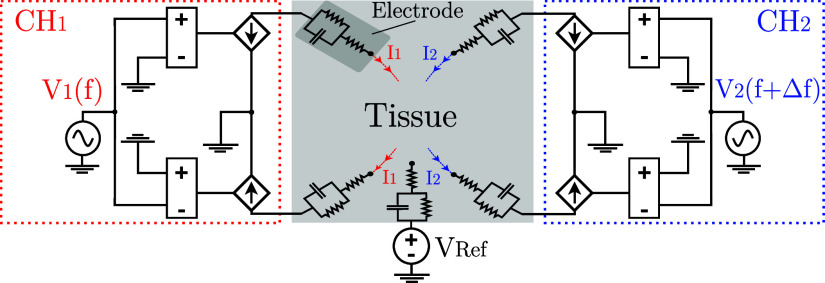
Schematic of an anti-phasic, two-channel tTIS stimulator, adapted from [5].

A tTIS stimulator requires a minimum of four electrodes in total (two electrodes per channel). To minimize crosstalk between channels, each channel implements an anti-phasic current drive, where two current sources per channel deliver signals to the tissue that are 180$^{\circ }$ out of phase (Fig. 3) [5]. This balanced configuration ensures equal but opposite-phase currents flow through each electrode pair, enabling precise stimulation control. A reference electrode biased at $V_{\mathrm{Ref}}$ can be used to sink imbalance currents, preventing charge accumulation.

**Fig. 4.**
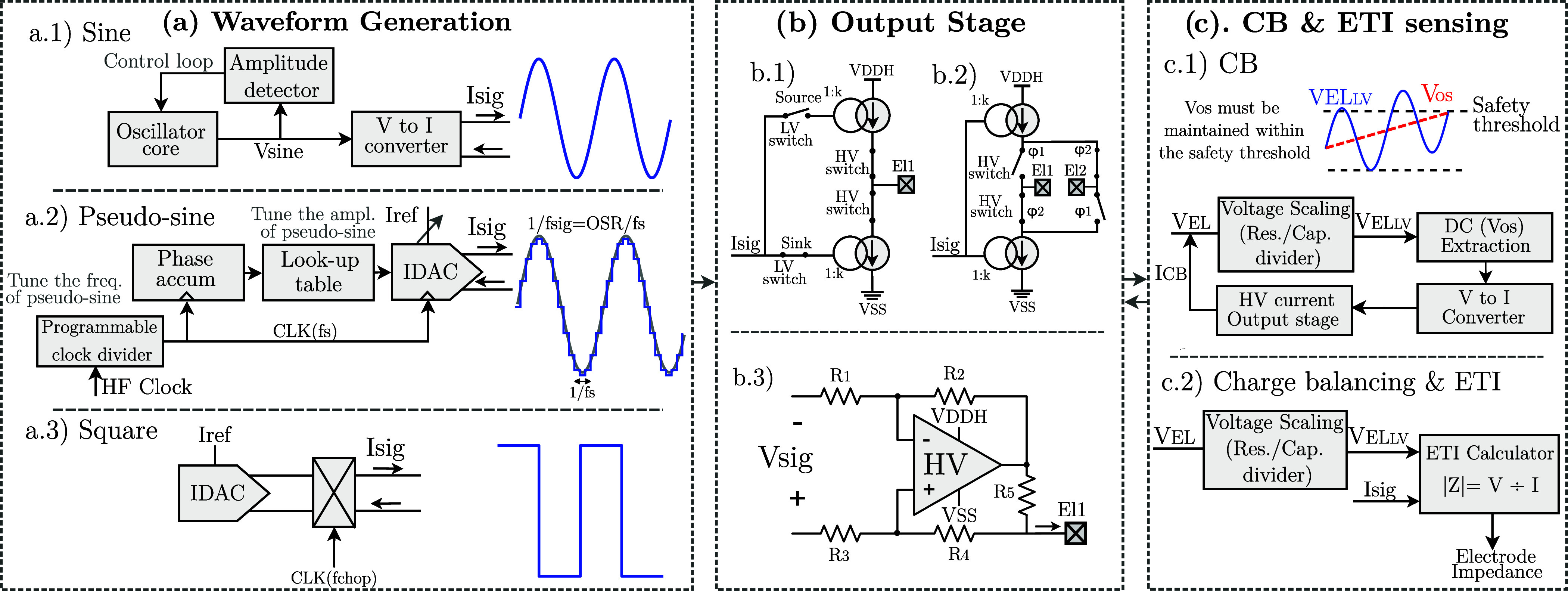
tTIS stimulator composed of: (a) waveform generation, (b) output stage, and (c) charge balancing (CB) and ETI sensing.

The circuit architecture of tTIS systems typically consists of three components: waveform generation, current injection, and charge balancing (Fig. 4).

### A. Waveform Generation

Accurate current waveform generation is critical for effective tTIS. The stimulation setup used in the original in vivo demonstration reported a total harmonic distortion (THD) below 0.4 [5], and later TI hardware implementations have likewise targeted low waveform distortion [25], [28]. Several on-chip waveform-generation methods reported for biomedical integrated circuits are relevant in this context [29], [30], [31]. Traditional analog oscillators generate high-linearity sinusoidal signals using reactive feedback loops (Fig. 4.a.1) [29]. However, their performance is highly sensitive to process, voltage, and temperature (PVT) variations and demands large capacitors at low frequencies. Direct Digital Synthesis (DDS) addresses these limitations by offering improved frequency control and generating pseudo-sinusoidal waveforms through current-steering Digital-to-Analog Converters (DACs), consolidating waveform synthesis and voltage-to-current conversion into a single stage (Fig. 4.a.2), as adopted in [25], [26], [27]. DDS introduces harmonic components at multiples of the pseudo-sinewave frequency $(k\,f_{\mathrm{psine}})$, together with spectral images around the sampling frequency $f_{s} \pm f_{\mathrm{psine}}$. By choosing a sufficiently high sampling rate, $f_{s}=\mathrm{OSR}\cdot f_{\mathrm{psine}}$ (with a large oversampling ratio $\mathrm{OSR}$), these components can be shifted well above the stimulation band, and attenuated by the low-pass characteristics of the electrode-tissue interface (ETI) [32], [33]. Alternatively, square-wave generation via Phase-Locked Loops (PLLs) and current-switching circuits (Fig. 4.a.3) offers greater power efficiency and lower design complexity. However, the resulting odd harmonics spread the signal energy over multiple frequency components. This issue is particularly critical in closed-loop systems (where stimulation and neural recording occur simultaneously [34]), as these harmonics can overlap with the neural signal band, degrading the recorded signal fidelity and complicating the suppression of stimulation artifacts.

### B. Output Stage

The output stage must drive the tissue with precise current waveforms while maintaining high output impedance, so that the delivered current remains stable despite variations in ETI impedance. It must also provide sufficient bandwidth for the carrier frequencies used in tTIS and enough voltage compliance to sustain the required current through high-impedance loads. In peripheral nerve stimulation, reported implementations span from a cuff-based rat interface operating at 3.3 V compliance and around 3 mA peak-to-peak current [26] to implantable peripheral nerve stimulators with 1022 V compliance and maximum currents around 10 mA [25], [27]. In transcranial applications, stimulation is generally delivered at low-mA levels, with many human studies using 13 mA [8], [9], [10], [11], [13], [14], [15], but substantially higher compliance voltages are required because of the higher ETI at the scalp. This is reflected in reported transcranial hardware requiring differential compliance on the order of tens of volts, from an ASIC stimulator providing 40 V differential compliance at 2 mA [28] to a discrete-component implementation providing 52 V differential compliance and up to 14 mA maximum current [35].

To meet these requirements, the waveform generated in the low-voltage domain must be translated into the high-voltage domain by an appropriate output-stage architecture. One common solution is a monopolar configuration with two independent current sources per electrode to source and sink current (Fig. 4.b.1), resulting in four current sources per tTIS channel [25], [27]. A second solution is a bipolar configuration based on a high-voltage H-bridge, which reduces the requirement to two current sources per channel by steering the current path through the electrode pair (Fig. 4.b.2). More recently, amplifier-based high-voltage current drivers have been investigated for tTIS, including implementations based on the Howland current pump topology (Fig. 4.b.3) [28]. In this architecture, a low-voltage stimulation waveform, typically generated by a voltage DAC, is converted into a controlled bidirectional output current while the amplifier provides the required voltage compliance. Under ideal resistor matching, $R_{2}/R_{1} = (R_{4}+R_{5})/R_{3}$, the output current is ideally $I_{\mathrm{El1}} = V_{\mathrm{sig}}/R_{5}$. Because the current is regulated by feedback rather than only by switching between current paths, this approach can improve linearity and reduce sensitivity to process, voltage, and temperature (PVT) variations.

### C. Charge Balancing and ETI Sensing

When delivering electrical stimulation, it is essential to avoid the accumulation of net charge at the ETI beyond safe thresholds. Ideally, stimulation waveforms should have perfectly matched positive and negative phases to ensure zero net charge transfer. However, in practice, achieving perfect symmetry is hindered by factors such as component mismatch and PVT variations in IC design. Even small imbalances can cause a DC voltage to build up over time, leading to irreversible electrochemical reactions, electrode corrosion, and potential tissue damage [36], [37]. To mitigate these effects, several neurostimulators employ active charge-balancing schemes that sense the residual electrode voltage during the interpulse interval and compensate for it in the following stimulation cycle [34], [38], [39], [40]. Although the acceptable residual voltage ultimately depends on the electrode material and ETI characteristics [36], [37], circuit implementations adopt residual-voltage targets in the low tens-of-millivolts range (commonly $\pm 50$ mV), while more stringent designs report regulation down to only a few millivolts [34], [39], [40], [41].

These techniques are not suitable for tTIS due to its semi-continuous operation, which can lead to unsafe charge buildup between extended stimulation cycles [25]. Similarly, passive DC-blocking capacitors placed in series with each electrode to remove the residual DC voltage are impractical for on-chip integration due to their large area requirements. To achieve real-time charge balancing without relying on large on-chip capacitors, TIS systems employ a multi-step process. Initially, the electrode voltage is reduced to a low-voltage range using a resistive or capacitive divider, enabling processing by standard low-voltage circuits. Next, the DC component of the electrode voltage is extracted through appropriate signal processing methods (Fig. 4.c.1). In [25], [27], this process is implemented by using peak detectors to estimate the mean electrode voltage. The extracted DC voltage ($V_{\mathrm{os}}$) is then converted into a compensation current by a transconductance amplifier, level-shifted to the high-voltage domain, and injected back into the electrode achieving residual offsets in the 3550 mV range.

The same voltage sensing circuit can also enable simultaneous ETI monitoring without additional hardware by calculating the absolute value of impedance from the measured electrode voltage and the known injected current (Fig. 4.c.2) [25]. Impedance data can be used to detect electrode connection issues and dynamically adjust stimulation parameters to maintain constant stimulation strength despite tissue changes.

## V. Application Challenges

Despite advances in computational modeling and animal studies, a comprehensive understanding of the mechanisms underlying tTIS remains elusive. When key parameters such as carrier frequency, stimulation amplitude, electrode size, and electrode placement are carefully controlled, tTIS has shown favorable tolerability and safety profiles [15]. However, deviations from these optimal conditions can lead to adverse effects, as excessive current amplitudes may induce intolerable skin sensation. To address this limitation, recent studies have proposed the use of carrier frequencies above 10 kHz, which can reduce skin sensation and may permit the use of higher stimulation amplitudes, although practical amplitude limits still apply [42], [43]. Importantly, recent evidence suggests that, although skin sensation decreases at higher carrier frequencies, amplitude-modulated TI can still retain a physiologically relevant component at $\Delta f$ capable of modulating neural activity [6], [12], [44]. Human intracranial studies are consistent with this view, showing that high-carrier sham stimulation without a frequency offset can be ineffective, whereas amplitude-modulated TI can still alter neural biomarkers [12], [44]. These findings are consistent with prior biophysical work suggesting that passive membrane low-pass filtering alone is insufficient to fully explain TI effects. However, whether TI in the human brain depends on a distinct amplitude-modulation-specific mechanism or on nonlinear responses that may also be shared with unmodulated kHz stimulation remains unresolved [6], [7], [12].

From an engineering perspective, increasing the carrier frequency can also reduce ETI impedance, thereby relaxing the voltage compliance requirements of the output stage [24]. At the same time, higher carrier frequencies may also reduce stimulation efficiency in the brain, which can complicate the trade-off between compliance requirements and neuromodulatory effectiveness [6].

Electrode placement, together with electrode size, determines the stimulation target in tTIS. However, amplitude imbalance between the two electrode pairs can unintentionally steer the interference envelope and thereby cause off-target activation. Although preliminary safety guidelines exist for current thresholds intended to prevent skin sensation, thermal effects, or undesired neural activation in humans [43], establishing quantitative safety limits for new tTIS architectures and modulation strategies remains a major challenge. Furthermore, findings from animal studies do not directly generalize to human applications because of interspecies differences in brain size, anatomy, and tissue conductivity [45], [46]. Accordingly, approaches such as mp-tTIS and m-tTIS require further validation to assess whether their improved focality and targeting flexibility can be reproduced in humans.

## VI. Future Directions

Next-generation tTIS systems are expected to be integrated into compact, high-channel-count ASICs that support multi-waveform capabilities for standard tTIS and pwm-tTIS, accurate phase control for sp-tTIS and pm-tTIS, and full compatibility with mp-tTIS and m-tTIS. This will enable simultaneous modulation of multiple brain regions, critical for treating complex neurological disorders, or enhance focality and stimulation amplitude at specific targets. Additionally, these systems will support a wide range of carrier frequencies, including those above 10 kHz, with fine frequency resolution (e.g., $\Delta f< 1$ Hz). Achieving high-voltage compliance across varying electrodetissue impedances will remain a key design requirement, alongside fully integrated charge-balancing methods that ensure safety throughout the stimulation period without requiring off-chip components. Additionally, advanced safety features will be essential. These include real-time monitoring of current source voltage compliance to ensure accurate current delivery, automatic disconnection in cases where stimulation exceeds predetermined frequency-dependent safety limits, and adaptive adjustment of stimulation parameters based on real-time ETI impedance measurements. Upon detecting electrode disconnection, the system will suspend stimulation and provide alerts until proper electrode contact is restored.

Patient-specific computational modeling frameworks, including emerging digital-twin or virtual-brain-twin approaches, may support individualized tTIS planning by predicting subject-specific electric-field distributions and helping tailor electrode montages, current ratios, carrier frequencies, and stimulation amplitudes [47]. Optimization and machine-learning approaches may further accelerate this personalization by improving the selection of electrode configurations and stimulation parameters [48], [49], [50]. Building on these advances, closed-loop, noninvasive neuromodulation devices with real-time EEG feedback may automatically adjust stimulation parameters, enabling more precise and personalized therapies [51]. In this context, advanced signal processing and adaptive filtering techniques will be essential to support neural recording despite artifacts from high-frequency carrier stimulation [52], [53]. Together with refined computational modeling, these developments may support the development of more standardized and individualized tTIS planning workflows that account for inter-subject variability [20]. Long-term, large-scale clinical studies and dedicated safety frameworks will be needed to establish robust clinical protocols and exposure guidelines [43]. Finally, advances in thermal management, power efficiency, and wireless connectivity will facilitate user-friendly neuromodulation systems for daily use.

## VII. Conclusion

Transcranial Temporal Interference Stimulation (tTIS) is an emerging noninvasive technique for deep brain modulation with transformative clinical potential. Recent advances in system designs, such as multipoint or multipolar tTIS, have significantly improved spatial resolution and flexibility. Furthermore, recent ASIC implementations have demonstrated the feasibility of miniaturized tTIS systems, enabling high-voltage compliance, on-chip waveform generation, precise current steering, and integrated charge balancing. Despite these advancements, challenges remain, particularly in the development of standardized safety protocols and personalized strategies. Future designs are expected to incorporate closed-loop control with EEG-based neural sensing, advanced artifact rejection, and machine learningdriven optimization of stimulation parameters. With these technological innovations and rigorous clinical validation, tTIS has the potential to revolutionize noninvasive neuromodulation across a broad range of clinical applications.
